# Future Habitat Shifts and Economic Implications for *Ophiocordyceps sinensis* Under Climate Change

**DOI:** 10.1002/ece3.71327

**Published:** 2025-04-23

**Authors:** Liangliang Chen, Hongfen Teng, Songchao Chen, Yin Zhou, Dan Wan, Zhou Shi

**Affiliations:** ^1^ School of Environmental Ecology and Biological Engineering Wuhan Institute of Technology Wuhan China; ^2^ College of Environmental and Resource Sciences Zhejiang University Hangzhou China; ^3^ School of Public Administration Zhejiang University of Finance and Economics Hangzhou China; ^4^ College of Resources and Environmental Sciences Tibet Agricultural and Animal Husbandry University Nyingchi China

**Keywords:** climate change, economic implications, ensemble model, *Ophiocordyceps sinensis*, suitable habitat, Tibetan Plateau

## Abstract

*Ophiocordyceps sinensis* is a vital and unique traditional medicine native to the Tibetan Plateau (TP) and its adjacent regions. Its habitat has significantly diminished in recent years due to commercial harvesting and climate change. Although studies on the habitat of *O. sinensis* have been conducted, the impact of climate change on its future habitat and economy remains unclear. This study utilizes a comprehensive dataset on *O. sinensis* occurrences and employs a multi‐model approach (constructed by Classification Tree Analysis [CTA], Flexible Discriminant Analysis [FDA], Generalized Boosted Model [GBM], Generalized Linear Models [GLM], Multivariate Adaptive Regression Splines [MARS], Random Forest [RF], and MaxEnt models) to simulate its potential suitable habitat distribution on the TP under current and future climate change scenarios (SSP1‐2.6 and SSP5‐8.5). Through this modeling process, we examined the primary environmental factors influencing its distribution. Our results indicated that China produces 91.9% of the world's *O. sinensis* , with over 82% of this production concentrated in Sichuan, Tibet, and Qinghai Provinces. Altitude, warmest quarter precipitation, coldest quarter mean temperature, and herbaceous vegetation cover accounted for 90% of the variation in the distribution of *O. sinensis* . The suitable habitat was primarily concentrated at altitudes of 3500–4500 m above sea level and was expected to shift to higher altitudes in the future. The predicted habitats under different emission scenarios vary. Under the low emission scenario (SSP1‐2.6), there was a slight increase in suitable habitat, with a 0.14% increase by the 2050s and a 0.65% increase by the 2100s. Conversely, under the high emission scenario (SSP5‐8.5), there was a notable decrease in suitable habitat, with a projected 4.32% reduction by the 2050s and a 5.34% reduction by the 2100s. Additionally, the production of *O. sinensis* was expected to increase by 0.2%–5.2% under SSP1‐2.6 and decrease by 0.5%–7.2% under SSP5‐8.5 in the main production areas in China. These findings provide a theoretical basis for the conservation and sustainable harvest of *O. sinensis* , which is crucial for future conservation efforts, maintaining ecological balance, and supporting the sustainable socio‐economic development of local communities.

## Graphical abstract

*Ophiocordyceps sinensis*, a key traditional medicine from the Tibetan Plateau, is losing habitat due to overharvesting and climate change. This study shows that China, producing 91.9% of the global supply, will see suitable habitats shift to higher altitudes. The economic impact includes a potential production increase under low emissions but a significant decrease under high emissions, threatening local economies.

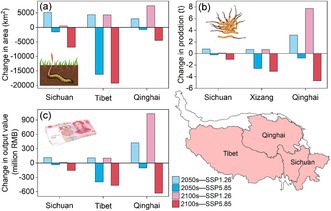

## 1 Introduction

The cordyceps fungus, *Ophiocordyceps sinensis*, is a valuable traditional remedy that has been used for more than 700 years and is commonly found on the Tibetan Plateau (TP) and its adjacent regions (Li et al. 2011; Yan et al. 2017). *O. sinensis* is called “*yartsa gunbu*” in Tibetan, which means “summer‐grass, winter‐worm,” although it is technically neither a grass nor a worm (Hu et al. 2005). It is a fungus that forms a complex with the underground larvae of ghost moths (Hepialidae), particularly those of the genus *Thitarodes*, by parasitizing them (Wang and Yao 2011). *O. sinensis* plays a regulatory role in the ecosystem by controlling the population of ghost moths, preventing overpopulation, and contributing positively to ecological balance. In this relationship, *O. sinensis* and the ghost moths are interdependent, but overharvesting or habitat destruction may disrupt this natural ecological balance (Liu et al. 2020). The primary bioactive components of *O. sinensis* are adenosine and cordycepin (Prasain 2013). It is not only a crucial ingredient in various health supplements and a valuable traditional medicine but has also been found to help treat many conditions, such as autoimmune diseases, high blood sugar, high cholesterol, low libido, cancer, chronic inflammation, fatigue, and type II diabetes (Olatunji et al. 2018; Yue et al. 2013).

The growing awareness of the medicinal and health benefits of *O. sinensis* , coupled with an expanding consumer base, has led to a yearly increase in demand (Tsering and Li 2012). This high demand and the limited distribution of the fungus have resulted in overharvesting, threatening local ecosystems and leading to its classification as an endangered species in China (Li et al. 2020). The distribution of *O. sinensis* is primarily influenced by natural environmental factors such as temperature, climate, and altitude (Wu et al. 2009; Yang et al. 2010). Several studies have quantitatively analyzed these factors. Vegetation cover, plant density, slope, predator presence, temperature, and humidity have been identified as key influences on its distribution (Wu et al. 2009). Temperature and precipitation are the primary meteorological factors affecting its yield (Chen et al. 2001). The distribution on the TP shows regional variations influenced by altitude, soil, climate, host plants, and topography (Yang et al. 2010). In Yushu, Qinghai Province, altitude, precipitation, slope, and aspect have been identified as important factors affecting its habitat (Li 2007). Due to variations in geographical regions and research focuses, results concerning the factors influencing *O. sinensis* show some variability. Therefore, a systematic analysis of environmental factors affecting the distribution of *O. sinensis* on the TP, integrating insights from previous research and various critical environmental elements, is necessary.

The primary distribution regions of *O. sinensis* are within the five provinces (regions) of the TP, with Qinghai and Tibet being the most significant (Li et al. 2011; Winkler 2008; Yang et al. 2010). The core distribution areas include Nagqu, Qamdo, and Nyingchi in Tibet; Yushu and Guoluo Tibetan Autonomous Prefectures in Qinghai; and Aba Tibetan‐Qiang Autonomous Prefecture in Sichuan. Approximately 80% of China's *O. sinensis* is produced in these regions (Yang et al. 2010). However, due to global climate change, the suitable habitats of many plants are altering (Salazar‐Tortosa et al. 2024; Van Daele et al. 2021). By the end of the 21st century, numerous plant species, especially those in mountainous regions, which are exceptionally sensitive to climate change, are expected to face severe threats, including potential extinction (Banerjee et al. 2024; Islam et al. 2023; Thuiller et al. 2005).

As one of the regions most vulnerable to climate change, 87% of the studied plant species on the TP are experiencing changes in their geographical ranges, shifting to higher altitudes (Chen et al. 2024; Choden et al. 2021; Telwala et al. 2013). The suitable habitats for *O. sinensis* on the TP and the surrounding areas have been noticed to be influenced by climate change (Hopping et al. 2018; Wei et al. 2021). There is a noticeable trend indicating a reduction in the distribution range of suitable habitats in the future, with an upward shift along the elevation gradient (Choden et al. 2021; Wei et al. 2021; Yan et al. 2017). However, some researchers propose contrasting views. Rising temperatures may shift suitable habitats to higher latitudes and altitudes on the TP, with moderate warming potentially expanding both the overall habitat area and regions suitable for high‐quality *O. sinensis* (Guo, Zhao, et al. 2021). In future climate change scenarios, the habitat area for this fungus in Nepal is projected to expand by 0.1%–4.9% (Shrestha and Bawa 2014). Therefore, the impact of climate change on the future habitats of *O. sinensis* remains uncertain. Accurate predictions of the future distribution patterns of suitable environments for this species under climate change are crucial for its successful preservation and sustainable harvesting, as well as for maintaining the stability of insect populations.

In this study, we utilized a comprehensive dataset and a reliable ensemble modeling approach to (1) distinguish the impacts of each variable and quantify their relative contributions to the distribution of *O. sinensis* , (2) model potential habitat distribution, and (3) predict its potential future trends under projected climate change scenarios. The findings provide a scientific basis for local governments to develop protection and regulatory frameworks, with significant implications for the conservation of *O. sinensis* , ecological balance, and sustainable socio‐economic development.

## 2 Materials and Methods

### 2.1 Study Area and Occurrence Data

**FIGURE 1:**
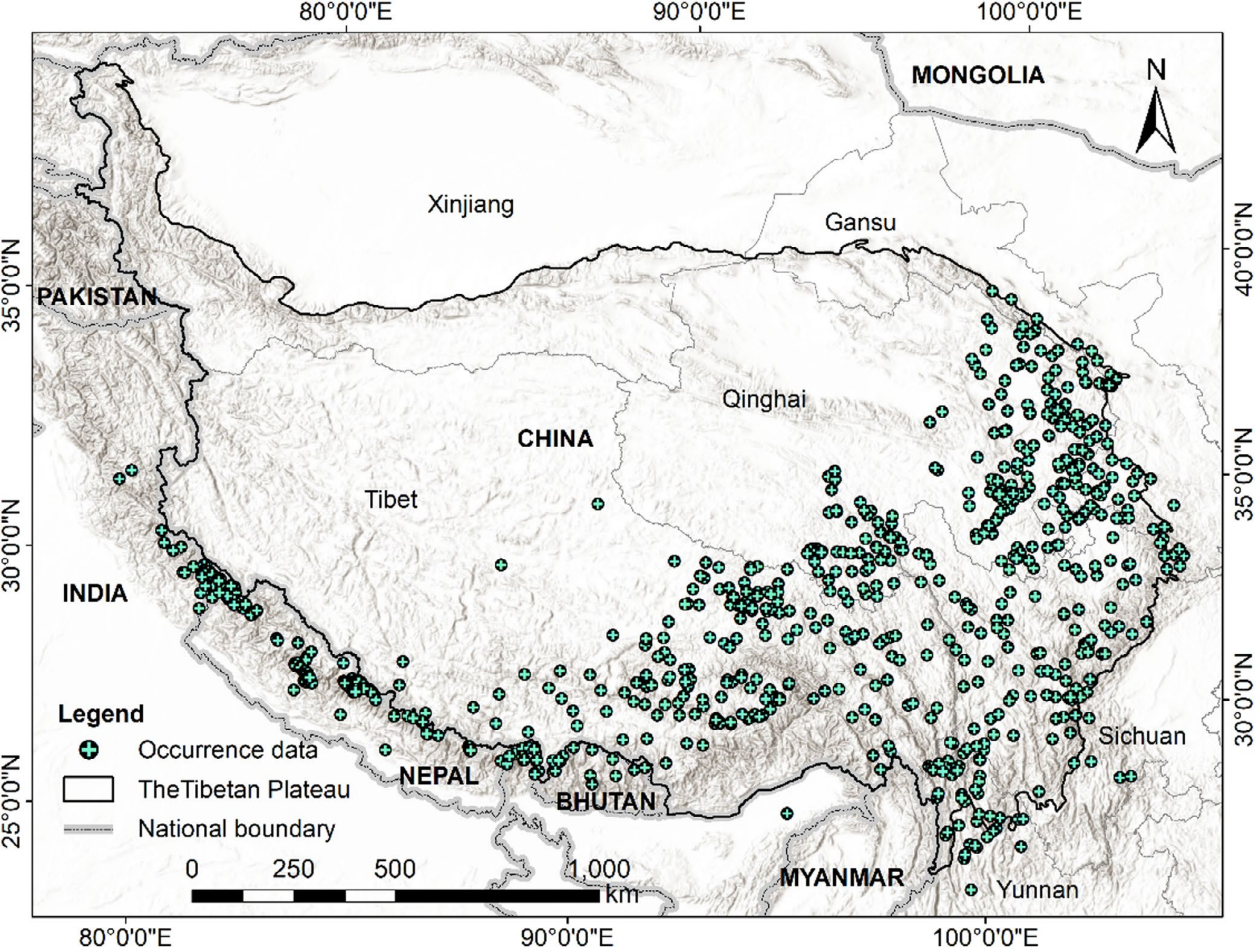
Study area and *Ophiocordyceps sinensis* occurrence data.

The TP, renowned for its *O. sinensis* production, was chosen as the main study area along with its surrounding regions. According to previous studies on suitable habitats for *O. sinensis* and its distribution data, these regions include northern Nepal, northern Bhutan, northeastern Pakistan, northern Myanmar, and northern India. In this study, we extensively collected occurrence data of *O. sinensis* by reviewing publicly available journal articles and relevant reports published both domestically and internationally. The primary sources were accessed through the PubMed database (https://pubmed.ncbi.nlm.nih.gov/). The search terms used included “*Ophiocordyceps sinensis*,” “*Cordyceps sinensis*,” and “Geographical distribution.” From the retrieved over 2000 articles, precise geographical coordinates of *O. sinensis* occurrence data were obtained, totaling 1442 records. These data were pre‐screened and validated by previous researchers to ensure high accuracy. Subsequently, duplicate records (469 occurrences) and wrong occurrences (five occurrences) were excluded. We also collected 24 occurrences of *O. sinensis* from the Global Biodiversity Information Facility (GBIF) (https://www.gbif.org/). The final dataset, comprising 968 unique occurrences, was used for subsequent analysis (Figure 1; Table S1).

### 2.2 Environmental Variables

We chose four sets of environmental variables that influence the growth of *O. sinensis* , including 10 soil variables, 19 historical bioclimatic variables, 9 land cover variables, and 3 topographic variables.

The topsoil datasets (0–30 cm) of gravel content, sand, silt, clay, USDA texture classification, organic carbon content, pH, calcium carbonate, gypsum, and sodicity (ESP) were downloaded from the Harmonized World Soil Database at 1 km resolution (HWSD v2.0, https://www.fao.org/soils‐portal/data‐hub/soil‐maps‐and‐databases/harmonized‐world‐soil‐database‐v20/en/, Guo, Zhao, et al. 2021). The 19 historical bioclimatic variables, representing the average of the years 1997–2000, were derived from the WorldClim v2.1 dataset at 1 km resolution (https://www.worldclim.org/) (Fick and Hijmans 2017). Elevation data were obtained from SRTM elevation data, and slope and aspect data were generated based on this elevation data. Land cover data were obtained from a global 1‐km consensus land‐cover product using generalized land‐cover classes that were provided by the EarthEnv project (Tuanmu and Jetz 2014) (https://www.earthenv.org/landcover/).

Future climate scenarios for the 2050s (averaged over the period 2040–2060) and the 2100s (averaged over the period 2080–2100) were derived from the CMIP6 future climate models. We selected seven global climate models (GCMs) from the CMIP6 project, including ACCESS‐CM2 (Australia), CMCC‐ESM2 (Italy), EC‐Earth3‐Veg (European), GISS‐E2‐1‐G (USA), HadGEM3‐GC31‐LL (UK), INM‐CM5‐0 (Russia), and IPSL‐CM6A‐LR (France). These models were selected because of the online data availability (https://www.worldclim.org/). The SSP1‐2.6 and SSP5‐8.5 climate scenarios, representing sustainable and fossil fuel‐rich development pathways, respectively, were utilized for future climate estimations (Forster et al. 2016). To be consistent with other environmental variables, all future climate data were set at a spatial resolution of 30 s.

To prevent dimensionality issues and overfitting, we used a workflow to select environmental variables. Initially, all variables underwent correlation analysis. Variables with a correlation coefficient below 0.6 were retained, while those exceeding this threshold were discarded. Next, the selected variables were categorized into four groups based on their characteristics. Each group underwent MaxEnt model predictions, iterated 10 times. Variables with a contribution rate exceeding 15% within each group were retained for further analysis. Subsequently, variables with a contribution rate above 15% underwent additional correlation analysis within each group to identify the most suitable variable. Following these steps, nine environmental variables were ultimately selected: Precipitation of Warmest Quarter (Bio18), Mean Temperature of Coldest Quarter (Bio11), Coverage of herbaceous vegetation (HV), Deciduous Needleleaf Trees_1 (NT), Topsoil silt fraction (T_SILT), Topsoil organic carbon (T_OC), Topsoil pH (H_2_O) (T_PH_H_2_O), elevation, and slope (Table 1). The correlation coefficients between these nine variables were all less than 0.6.

**TABLE 1:** Environmental variables used for modeling.

Factor	Variable name	Code	Data source
Bioclimatic	Mean Temperature of Coldest Quarter	Bio11	Worldclim
	Precipitation of Warmest Quarter	Bio18	Worldclim
Soil	Topsoil silt fraction	T_SILT	HWSD v1.2
	Topsoil organic carbon	T_OC	HWSD v1.2
	Topsoil pH (H_2_O)	T_PH_H_2_O	HWSD v1.2
Terrain	Elevation	ELE	SRTM
	Slope	SLOP	
Land cover	Coverage of herbaceous vegetation	HV	Earthenv
	Deciduous Needleleaf Trees	NT	Earthenv

### 2.3 Socioeconomic Data

Although *O. sinensis* is primarily found on the TP, its harvest and trade are regulated by local natives, making it difficult to track production and trade volumes by country. In this study, we collected socioeconomic statistical data from the main production areas of *O. sinensis* : the Tibet Autonomous Region, Qinghai Province, and Sichuan Province in China. These regions account for over 80% of its habitat and 90% of its production in China. The prices and production amounts were obtained from local government websites.

### 2.4 Current Potential Distribution Model

We used 10 single‐species distribution models to simulate the potential range of *O. sinensis* . These models include Classification Tree Analysis (CTA) (Edwards et al. 2006), Flexible Discriminant Analysis (FDA) (Hastie et al. 1994), Generalized Boosted Model (GBM) (Friedman 2001), Generalized Linear Models (GLM) (McCullagh 2019), Multivariate Adaptive Regression Splines (MARS) (Elith and Leathwick 2007), Maximum Entropy (MaxEnt) (Phillips et al. 2006), Random Forest (RF) (Mi et al. 2017), Generalized Additive Model (GAM) (Guisan et al. 2002), Surface Range Envelope (SRE), and Artificial Neural Network (ANN) (Linderman et al. 2010).

During model execution, both species presence data and pseudo‐absence data are required. Pseudo‐absence data were randomly generated within the study area using random sampling methods to replace true absence data. This method ensures broad environmental coverage and reduces potential biases associated with subjective selection criteria (Barbet‐Massin et al. 2012; Wisz and Guisan 2009). While elevation is an important factor influencing species distribution, restricting pseudo‐absence selection based solely on elevation could overlook other key environmental variables such as temperature and precipitation, potentially reducing model generalizability. To minimize sampling uncertainty and improve model robustness, we implemented two refinements: (1) Two independent sets of pseudo‐absence data, each containing 1000 points, were generated to enhance model stability; (2) each algorithm was run five times, and the results were averaged to reduce biases introduced by single‐instance pseudo‐absence selection.

The calibration area of the model was set to the TP and its surrounding regions, including northern Nepal, northern Bhutan, northeastern Pakistan, northern Myanmar, and northern India. For each single model algorithm, 80% of the data was used for training and 20% for validation. To enhance model reliability, a total of 100 individual model outputs (2 sets of pseudo‐samples × 10 model algorithms × 5 replicates) were generated. The importance of each environmental variable was calculated, with a higher score indicating greater importance and a score of 0 indicating no importance.

We evaluated the model accuracy using the area under the receiver operating characteristic curve (AUC) and the true skill statistic (TSS) (Eskildsen et al. 2013). AUC ranges from 0.5 (random classification) to 1 (perfect classification), with values above 0.8 indicating good performance and below 0.7 indicating poor performance (Bell and Fielding 1997). TSS ranges from 0 to 1, with values above 0.7 reflecting good predictive accuracy and values below 0.5 indicating poor accuracy (Guo, Jiang, et al. 2021).

**FIGURE 2:**
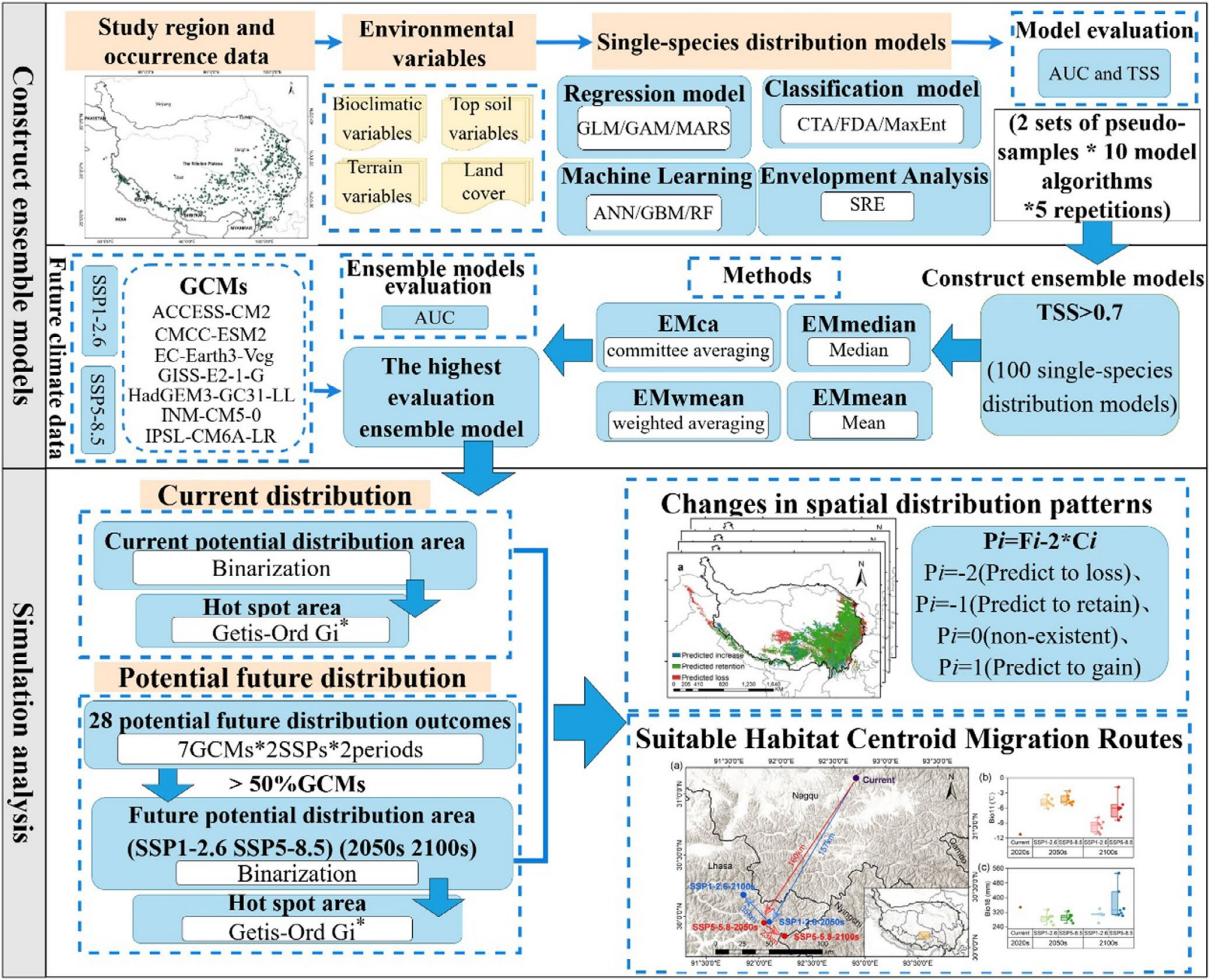
Flowchart of the method to predict the current habitat and its future changes of *O. sinensis* .

From the 100 runs of single model outcomes, we selected models with a TSS value greater than 0.7 and variables with an average importance greater than 0.1 for inclusion in the final ensemble model. The ensemble models were constructed using the mean (EMmean), median (EMmedian), committee averaging (EMca), and weighted averaging (EMwmean) methods based on these selected models. Ultimately, the ensemble model with the highest evaluation result was chosen to simulate the current and future suitable habitat distribution of *O. sinensis* . To visually display the predicted habitat distribution, the continuous suitability index was transformed into binary results (presence/absence) based on the receiver operating characteristic (ROC) threshold (Thuiller et al. 2009). The flowchart for building the ensemble model is shown in Figure 2.

### 2.5 Potential Future Distribution Model

To forecast the potential future range of *O. sinensis* , we employed seven GCMs from the CMIP6 project. Using a combination model, we projected the future range trends of its habitat under each climate model for two future time periods (2050s and 2100s) and two Shared Socioeconomic Pathway scenarios (SSP1‐2.6 and SSP5‐8.5). The environmental variables used were consistent with those employed to simulate the current potential range of *O. sinensis* , except for the historical bioclimatic variables. Consequently, we generated a total of 28 predicted outcomes, representing the results for each time period, SSP scenario, and GCM. Following this, if a consensus was reached among more than half of the GCM results, we produced maps depicting the projected alterations in suitable habitat for *O. sinensis* under the SSP1‐2.6 and SSP5‐8.5 scenarios for the 2050s and 2100s.

It should be noted that the dispersal ability of *O. sinensis* is highly reliant on its host, the ghost moth larvae, which significantly influences its distribution dynamics. These larvae live in soil at depths of 5–25 cm in alpine meadows, feeding on plant roots and stems, with a lifespan of 3–4 years or longer (Wang and Yao 2011). Both larvae and adult moths have limited dispersal abilities due to their restricted activity range and short lifespan (Qiu et al. 2016). Consequently, we only consider the no‐dispersal scenario for the future distribution prediction of suitable habitats for *O. sinensis* .

### 2.6 Hot Spot Area and Centroid

In this study, the Getis‐Ord Gi* statistic was used to identify the statistically significant spatial clusters of hot spots for *O. sinensis* . This method automatically aggregates occurrence data, determines an appropriate scale of analysis, and corrects for both multiple testing and spatial dependence (Getis and Ord 1992). Using this method, an output feature class of confidence level bins (Gi_Bin) was generated. In this study, the features with a Gi_Bin value of 3 (reflecting a 99% confidence level) were identified as its hot spots.

The centroid of suitable habitats was calculated for both the current and future predictions of this fungus. The centroid, which refers to the balance point of a species' distribution within a specific region at a given time, reflects the central position of its habitat and serves as an important indicator of geographical distribution (Lei et al. 2012; Wang et al. 2024). Changes in the centroid can reflect the spatial aggregation and migration of the species over time. Future climate change will lead to changes in the geographic distribution range of species, and the centroid of suitable habitats will also shift accordingly, resulting in the migration trajectory of the species' centroid. The migration of the centroid reflects the changes in species distribution, and the direction of change indicates the evolutionary direction of species redistribution within the region (Zhang et al. 2024). This study treated the suitable habitat patches as a whole to explore the direction and distance of centroid shifts under different future climate scenarios and time periods.

## 3 Results

### 3.1 Model Performance

**FIGURE 3:**
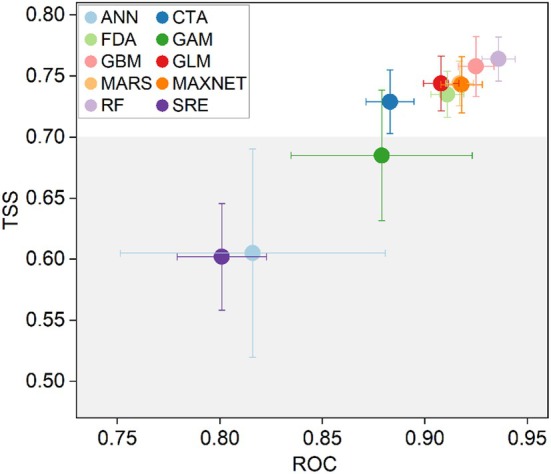
Accuracy of prediction results of different models.

The results of the AUC and TSS for the 10 species distribution models are presented in Figure 3. Among the models, RF demonstrates the highest accuracy and stability, with an average TSS of 0.76 and an average AUC of 0.94. GBM exhibits the next best performance, with average TSS and AUC values of 0.76 and 0.93, respectively. Conversely, SRE performs less favorably, with average TSS and AUC values of 0.60 and 0.80, respectively. Based on these results, we selected models with TSS values greater than 0.7 to construct the ensemble model, which are CTA, FDA, GBM, GLM, MARS, RF, and MaxEnt. Based on the performance of ensemble models, the best‐performing ensemble model, EMca, was used to predict the current and future suitable habitats of *O. sinensis* in the following analysis (Table 2).

**TABLE 2:** Evaluation metrics of ensemble models.

Name	AUC	TSS	Sensitivity	Specificity
EMca	0.98	0.87	87.69	99.50
EMmean	0.96	0.76	84.92	94.12
EMmedian	0.94	0.75	83.47	95.30
Emwmean	0.96	0.76	85.02	94.12

**FIGURE 4:**
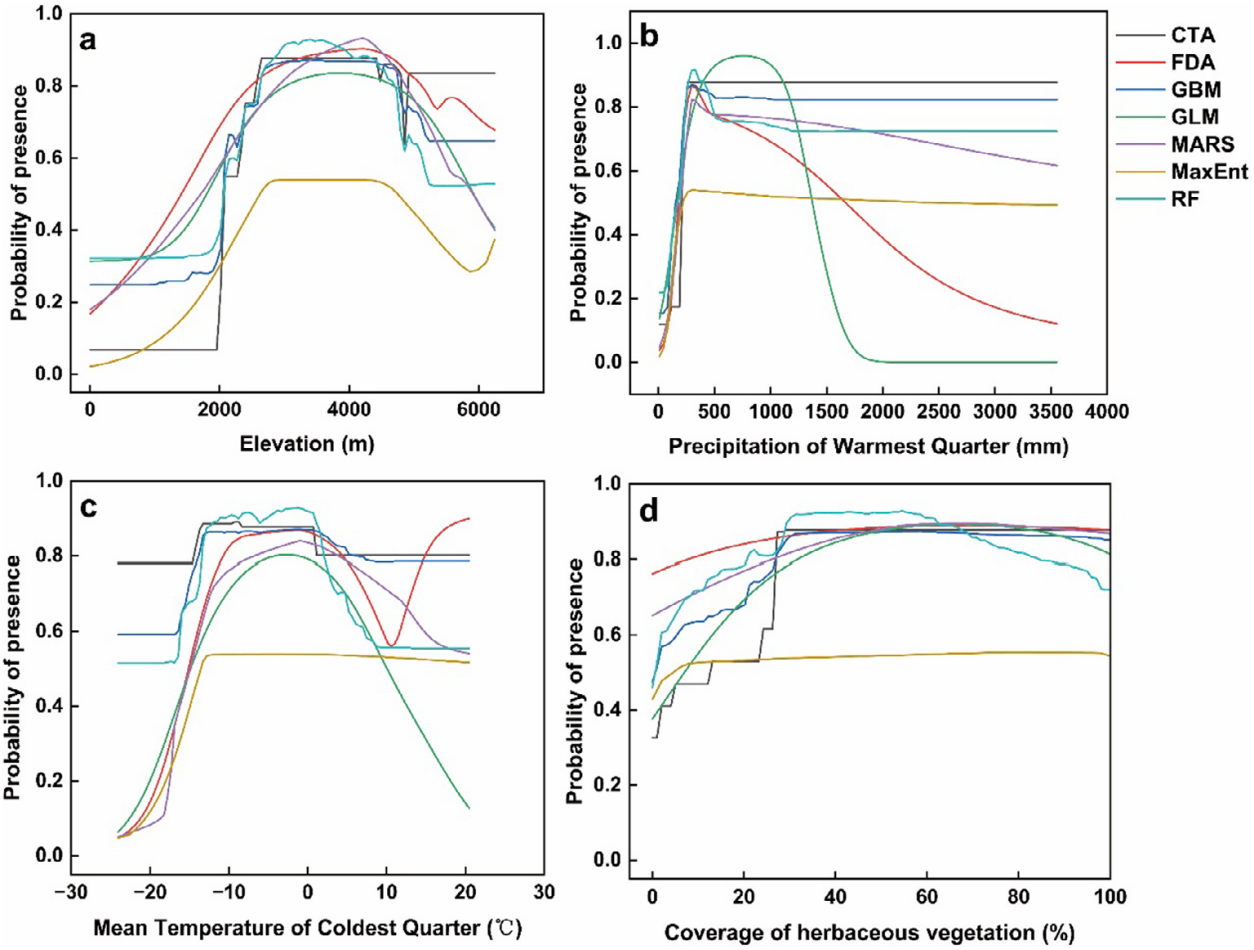
Response curves for the four critical environmental factors. (a) Elevation; (b) precipitation of warmest quarter; (c) mean temperature of coldest quarter; (d) coverage of herbaceous vegetation.

The response curves demonstrate how the predicted probability of occurrence varies with changes in elevation, Bio18, Bio11, and HV. Among the terrain variables, elevation plays a primary role in determining the distribution of *O. sinensis* . The species tends to occur between 3000 and 5000 m, with the highest occurrence probability observed within the range of 3000–4000 m (Figure 4a). In terms of precipitation gradient, the highest probability of occurrence is found within the range of approximately 100–300 mm (Figure 4b). *O. sinensis* predominantly occurs at average winter temperatures ranging from −15°C to 20°C, with the highest probability observed around −10°C to 4°C (Figure 4c). Among the land cover variables, herbaceous vegetation coverage is the main influencing factor, with suitable coverage being above 30% (Figure 4d).

### 3.2 Current Distribution of *O. sinensis*

**FIGURE 5:**
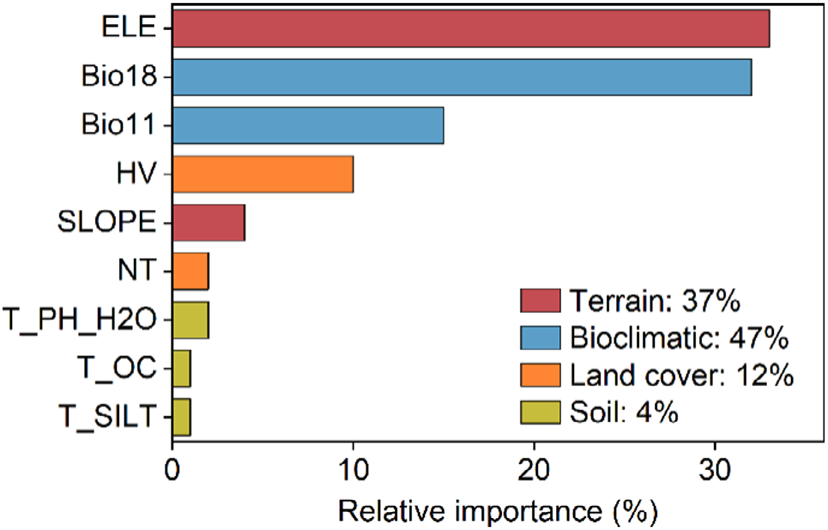
The importance of environmental variables.

According to the final ensemble model, ELE, Bio18, Bio11, and HV emerged as the four most significant environmental variables impacting the spatial distribution of *O. sinensis* , with relative importance values of 33%, 32%, 15%, and 10%, respectively (Figure 5). The cumulative contribution of these four environmental variables reached 90%, underscoring their pivotal role in shaping its distribution. Furthermore, bioclimate was the most important variable, explaining 47% of the spatial occurrence of *O. sinensis* . The contribution of terrain was comparable to other factors, with a relative importance of 37%. Soil showed the lowest relative importance, at 4%, among the environmental variables (Figure 5).

**FIGURE 6:**
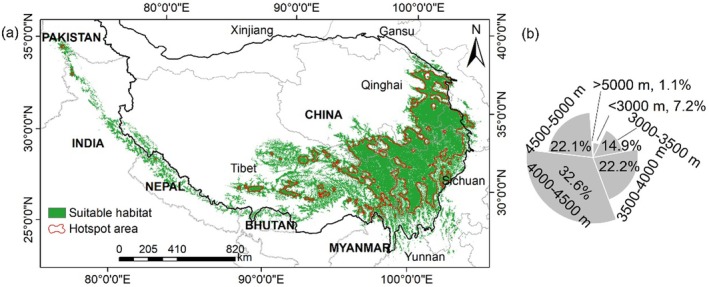
(a) Spatial distribution of the predicted current habitat suitability of *O. sinensis* . (b) The insert figure shows the ratio of suitable habitat among elevation ranges.

The predicted current potential distribution of *O. sinensis* based on the ensembled model is shown in Figure 6. The estimated potential distribution area is approximately 75.5 × 10^4^ km^2^, primarily situated in the southwestern part of the TP within China, northern Nepal, northern Bhutan, northeastern Pakistan, Myanmar, and northern India. Within these countries, China hosts the most extensive suitable distribution area, accounting for 91.9%, followed by India (3.1%), Nepal (2.9%), Pakistan (0.8%), Bhutan (0.8%), and Myanmar (0.5%) (Table 3). The hot spot area is 37.24 × 10^4^ km^2^, nearly half of the total potential distribution area, predominantly within the TP. According to the prediction, 91.7% of its suitable habitat is within the elevation range of 3000–5000 m, with 7.2% below 3000 m and 1.1% above 5000 m. The elevation range of 4000–4500 m holds the most suitable habitats (32.6%), followed by 3500–4000 m and 4500–5000 m, each holding about 22% (Figure 6). The results of current habitat suitability based on the ensemble model are shown in Appendix A.

**TABLE 3:** Current suitable area of *O. sinensis* in different countries.

Country	Province	Suitability area (10^4^ km^2^)	Proportion (%)
China	Sichuan	20.90	27.7
	Guizhou	0.01	0.0
	Yunnan	2.31	3.1
	Xizang	26.63	35.2
	Gansu	5.19	6.9
	Qinghai	14.39	19.0
	Total	69.43	91.9
Nepal		2.20	2.9
Bhutan		0.58	0.8
Pakistan		0.61	0.8
Myanmar		0.37	0.5
India		2.37	3.1
Total		75.56	100.0

### 3.3 Potential Future Distribution of *O. sinensis*

**FIGURE 7:**
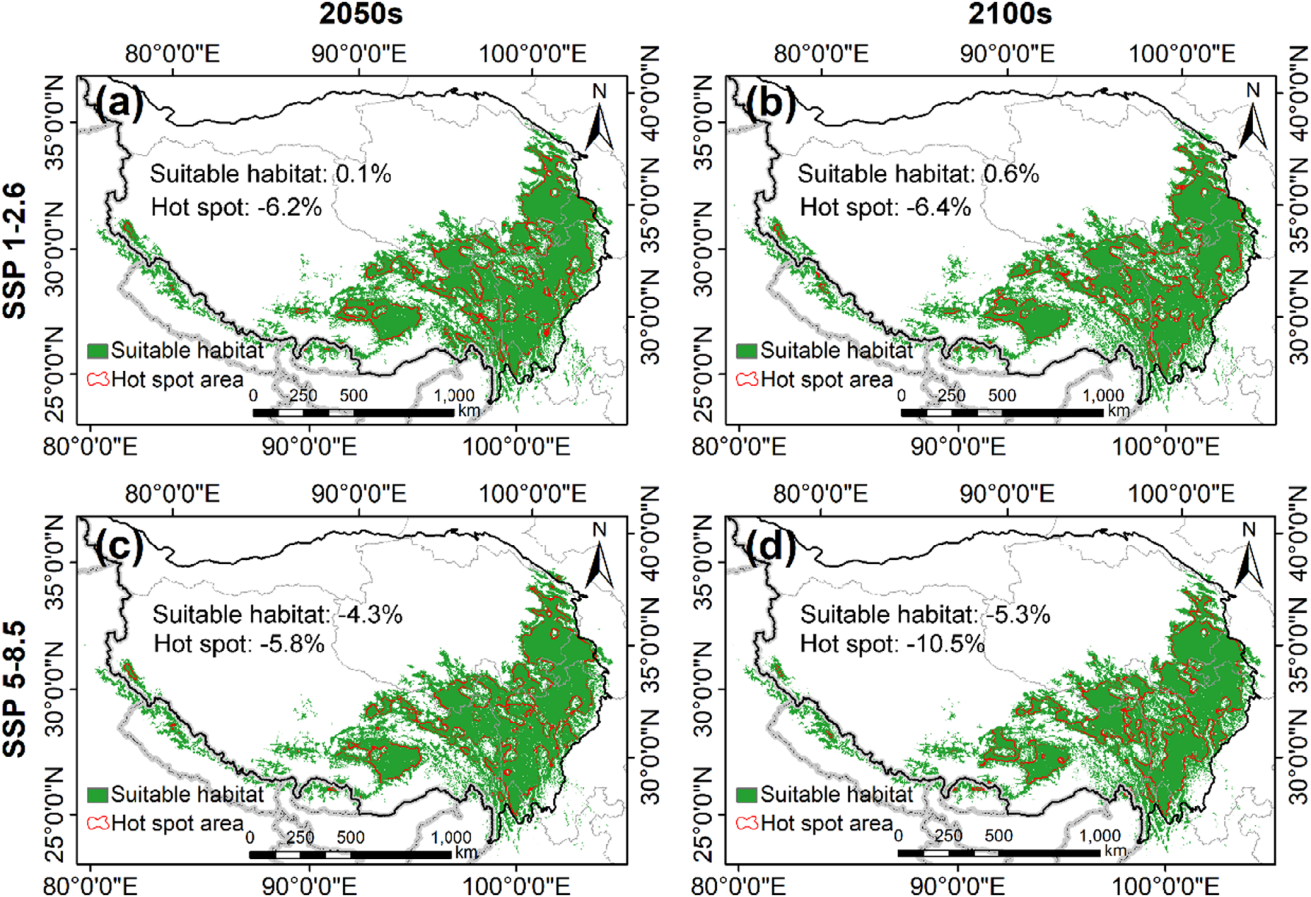
The habitat suitability of *O. sinensis* under different climate scenarios. (a) SSP1‐2.6 in the 2050s; (b) SSP1‐2.6 in the 2100s; (c) SSP5‐5.8 in the 2050s; (d) SSP5‐8.5 in the 2100s.

Consistent with the simulation of the current potential distribution, we further employed the ensemble model to forecast the potential future range of *O. sinensis* under four climate change scenarios (two SSPs and two time periods). According to Figure 7, the suitable habitats for *O. sinensis* vary under different scenarios compared to the current potential habitat. Under SSP1‐2.6, suitable habitat increases slightly by the 2050s and 2100s, with increases of 0.1% and 0.6%, respectively (Figure 7a,b). However, the corresponding hot spot areas decreased by more than 6%. Under the high‐forcing SSP5‐8.5 scenario, suitable habitat decreases by 4.3% by the 2050s and 5.3% by the 2100s (Figure 7d). Additionally, suitable habitat in Pakistan and India, as well as near the Qilian Mountains in Gansu Province, China, will disappear in the future. The uncertainties of habitat suitability for *O. sinensis* under different climate change scenarios are shown in Appendix B.

**FIGURE 8:**
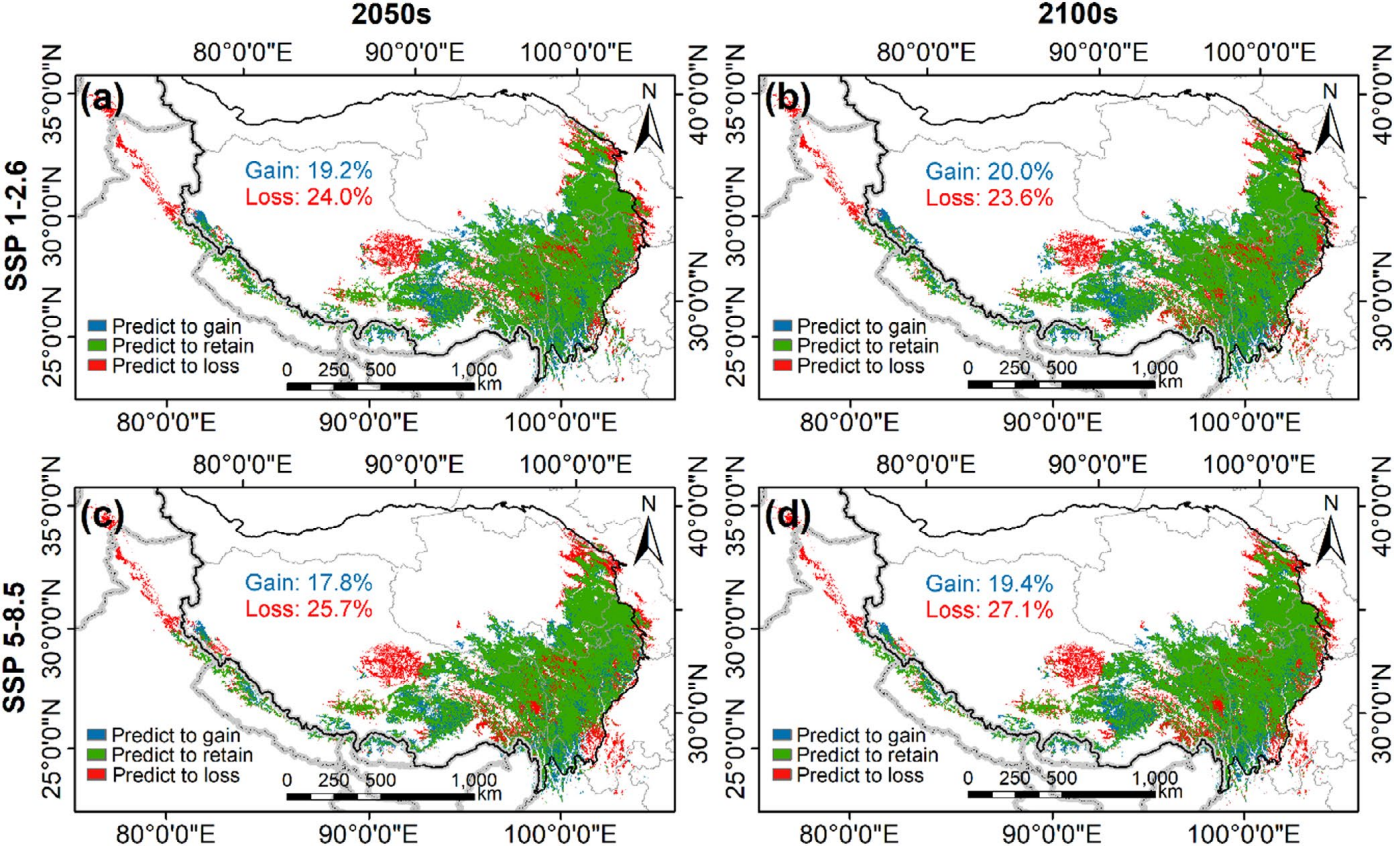
The changes in spatial distribution patterns of *O. sinensis* under different climatic scenarios. (a) SSP1‐2.6 in the 2050s; (b) SSP1‐2.6 in the 2100s; (c) SSP5‐5.8 in the 2050s; (d) SSP5‐8.5 in the 2100s.

By comparing the predicted current and future suitable habitat, we observe that the augmented suitable habitat primarily spans areas in the Tibet Autonomous Region, Yunnan Province, Qinghai Province, and Sichuan Province. In contrast, the most suitable lost habitat is mainly located in Pakistan, India, Nagqu City in the Tibet Autonomous Region, and the eastern edge of the TP (Figure 8). The SSP5‐8.5 scenario indicates a greater loss of suitable habitat compared to the SSP1‐2.6 scenario (25.7% vs. 24.0% in the 2050s, 27.1% vs. 23.6% in the 2100s). Conversely, the gain of suitable habitat in the SSP5‐8.5 scenario is less than that in the SSP1‐2.6 scenario (17.8% vs. 19.2% in the 2050s, 19.4% vs. 20.0% in the 2100s).

**FIGURE 9:**
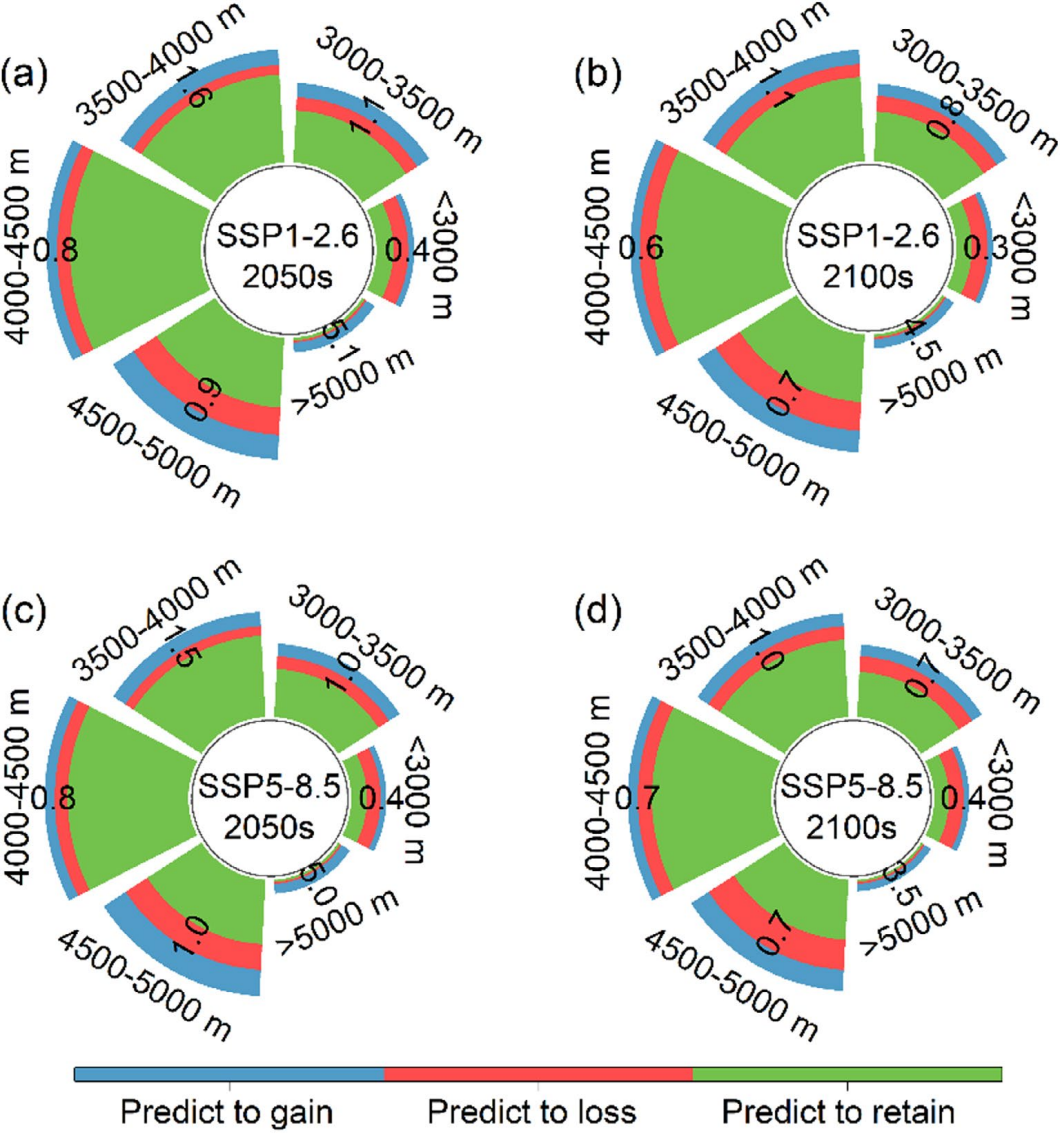
The changes in spatial distribution patterns of *O. sinensis* under different climatic scenarios. (a) SSP1‐2.6 in the 2050s; (b) SSP1‐2.6 in the 2100s; (c) SSP5‐5.8 in the 2050s; (d) SSP5‐8.5 in the 2100s. The numbers in the figures show the ratio of gain to loss.

We predicted that the suitable habitat for *O. sinensis* will exhibit significant variations among different elevation ranges in the future. Generally, the majority of suitable habitats are concentrated in the range of 4000–4500 m (Figure 9). Under SSP1‐2.6, there is a noticeable increase in habitat above 5000 m, with a gain‐to‐loss ratio of 5.1 in the 2050s and 4.5 in the 2100s. However, habitats below 3000 m showed a significant decrease, with a gain‐to‐loss ratio of 0.4 in the 2050s and 0.3 in the 2100s (Figure 9a,b). A similar trend was detected under SSP5‐8.5. Overall, in the 2100s, there is a decrease in the added habitat compared to the 2050s, along with an increase in lost habitat, mainly observed between 4500 and 5000 m. Regardless of SSP1‐2.6 or SSP5‐8.5, habitats above 5000 m increased compared to the current situation (Figure 9).

### 3.4 Future Suitable Habitat Centroid Migration Routes

**FIGURE 10:**
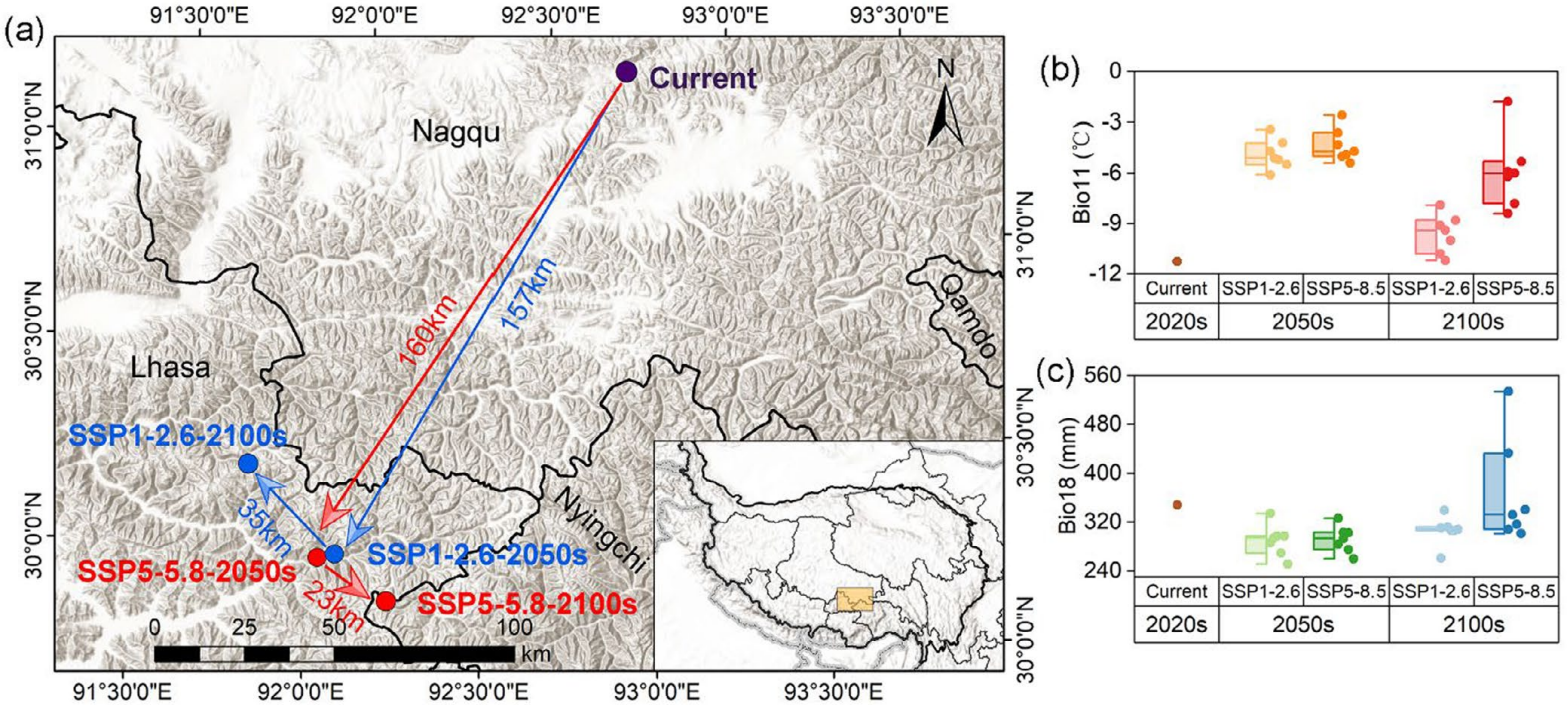
Changes of suitable habitat centroid of *O. sinensis* under different climate change scenarios in the 2050s and 2100s. (a) Future suitable habitat centroid migration pathway. The main climate variables of (b) the Bio11 and (c) the Bio18 for the current and future centroids.

In the current period, the centroid of the suitable habitat for *O. sinensis* is located in Nagqu City, Tibet Autonomous Region (Figure 10a). With global warming and increased humidity, the centroid of the suitable habitat is projected to shift southwestward by approximately 160 km in the 2050s. Specifically, it moves to the east of Lhasa, Tibet Autonomous Region, under both SSP1‐2.6 (157 km) and SSP5‐8.5 (160 km). However, with further climate changes in the 2100s, the suitable habitat centroid may shift in two opposite directions. Under SSP1‐2.6, the centroid moves north of Lhasa (35 km), while under SSP5‐8.5, it migrates toward Nyingchi (23 km) (Figure 10a).

The comparison of Bio11 and Bio18 values for the current centroid and its future locations is shown in Figure 10b,c. Bio11 at the centroids for current and future experienced a trend of first increasing and then decreasing, while Bio18 experienced a trend of first decreasing and then increasing. Specifically, the current centroid of the suitable habitat for *O. sinensis* has a Bio11 value of −11°C. In the 2050s, the centroids have a similar mean Bio11 of about −4.5°C. By the 2100s, the mean Bio11 value decreased to −5.9°C and −9.6°C for SSP1‐2.6 and SSP5‐8.5, respectively (Figure 10b). The current centroid has a Bio18 value of 348 mm. In the 2050s, the centroids have a similar mean Bio11 of about 290 mm, while in the 2100s, the mean Bio18 value increased to 306 and 360 mm for SSP1‐2.6 and SSP5‐8.5, respectively (Figure 10c).

**FIGURE 11:**
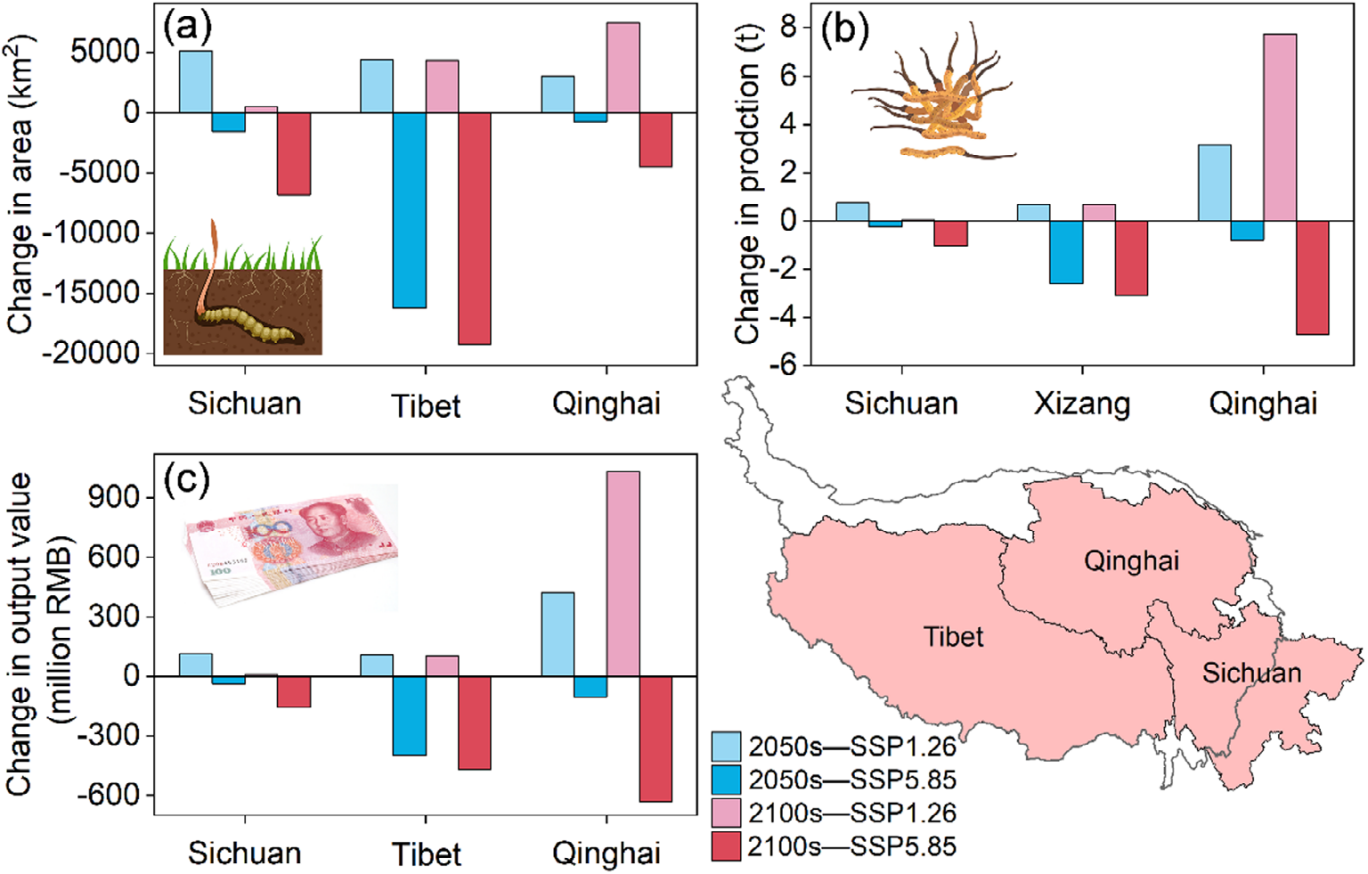
Future changes of (a) habitat area, (b) production, and (c) output value in Sichuan, Tibet, and Qinghai Province, China, under the scenario of SSP1‐2.6 and SSP5‐8.5 for the 2050s and 2100s.

China produces 91.9% of the world's *O. sinensis* , with Sichuan, Tibet, and Qinghai Province accounting for over 82% of this production. According to local government websites, the current production in these three provinces is 31.5, 42.4, and 150 tons, respectively. Figure 11a illustrates the changes in habitat area in these provinces. This study provides a rough estimate of the future production of *O. sinensis* under climate change based on the existing production data and the corresponding suitable habitat area in these three provinces. The habitat areas in these provinces increase in the 2050s and 2100s under SSP1‐2.6, especially in Qinghai Province in the 2100s (7410 km^2^). Tibet shows the most noticeable decrease under SSP5‐8.5 (−16,220 km^2^). With future climate change, production in these three provinces shows an increasing trend under SSP1‐2.6 and a decreasing trend under SSP5‐8.5 (Figure 11b). Qinghai Province shows the most obvious change in the 2100s under SSP1‐2.6 (7.7 tons) and SSP5‐8.5 (−4.7 tons). Correspondingly, in terms of output value, Qinghai Province shows the most obvious decrease in the 2100s under SSP5‐8.5 based on the current market value (−630 million RMB) (Figure 11c).

## 4 Discussion

In the past, predicting potential distributions of species has mostly relied on single‐species distribution models (Xu et al. 2015). However, different species distribution model algorithms have their own advantages and limitations, and no single model algorithm can best simulate all species distributions in every study (Qiao et al. 2013). Therefore, no optimal model is suitable for all species. A single model has limited generalization ability and is more suitable for simple problems. A single model cannot better simulate a complex fungal species like *O. sinensis* . To reduce the bias of a single model and obtain more reliable predictions, in this study, we comprehensively considered the impacts of climate, topography, soil, and vegetation on the suitable habitat for *O. sinensis* with an ensemble model. The potential range of this fungus predicted by the composite model closely aligns with its known survival range and geographical distribution reports (Li et al. 2011). The predicted potential distribution area primarily spans the southwestern part of the TP, including regions in China, Nepal, Bhutan, northern India, and Pakistan, with China holding the most extensive suitable area (91.9%). This finding is consistent with existing literature, emphasizing the ecological importance of the TP as a key habitat for this fungus (Guo, Zhao, et al. 2021; Li et al. 2020; Wei et al. 2021). Future climate scenarios predict significant changes in the suitable habitats for *O. sinensis* . Under SSP1‐2.6, suitable habitats slightly increase, whereas under SSP5‐8.5, they decrease notably.

### 4.1 Main Factors Influencing the Distribution of *O. sinensis*

Our results indicated that elevation, Bio18, Bio11, and HV are the key variables influencing the spatial distribution of *O. sinensis* , contributing a cumulative 90%.

#### 4.1.1 Elevation

Elevation significantly impacts its distribution, with 91.7% of suitable habitats located between 3000 and 5000 m, particularly between 4000 and 4500 m (32.6%). This preference for high‐altitude environments is supported by previous research and is likely due to specific climatic conditions such as lower temperatures and higher humidity (Chen et al. 2001; Wu et al. 2009; Yang et al. 2010; Zhang et al. 2011). Notably, suitable habitats below 3000 m are gradually diminishing, while those above 5000 m are increasing, indicating an upward shift in altitude for suitable habitats. Previous studies have demonstrated a trend of suitable habitats migrating to higher altitudes. For example, in the 1960s, the lower limit of distribution altitude in the core distribution area of *O. sinensis* in Nagqu, Tibet, was 3500 m (Diao 1996), which rose to 4100 m by the 1990s (Liu and Li 2006). In Yushu, Qinghai Province, the lower limit of distribution altitude was 3600 m in the 1960s, rising to 4000 m by the 1980s (Sheng et al. 1988). This trend may be related to the rising snow line on the TP due to climate warming (Zhang et al. 2010), leading to a decrease in soil moisture content, thereby affecting vegetation and the survival of ghost moth larvae. Studies have shown that climate warming has led to earlier insect phenology and a shift in their geographical distribution toward higher latitudes and altitudes. This could also be the reason why *O. sinensis* may move to higher altitudes in the future (Chen and Ma 2010).

#### 4.1.2 Bioclimatic

The cumulative contribution of Bio 18 and Bio 11 reached 47%, highlighting summer precipitation and winter temperature as the major factors influencing *O. sinensis* distribution. Precipitation has a significant impact on its growth, development, and yield (Zhang et al. 2011). The optimal habitat occurred within the range of 200–500 mm for summer precipitation (Figure 4b); excessively high or low precipitation levels were detrimental to its growth. For example, the main production areas in eastern Nagqu, such as Suoxian, Biru, Baqing, and Jiali, receive 250–500 mm of precipitation, which is significantly higher than the 200 mm received in non‐main production areas like Nierong County and various western counties (Chen et al. 2001).

Winter temperature is another critical factor for the distribution of *O. sinensis* , affecting its survival and development. Previous studies indicated that within a certain temperature range, insect development rates tend to increase with rising temperatures (Damos and Savopoulou‐Soultani 2012). Optimum habitat suitability for *O. sinensis* is observed when the winter average temperature ranges from 10°C to 5°C, with suitability declining as temperatures exceed 5°C (Figure 4c). The production region's climate features a distinct division between a prolonged winter half‐year with lower average temperatures and a summer half‐year with comparatively higher monthly average temperatures (Zhang et al. 2011). The growth of *O. sinensis* begins at an average temperature of 2.6°C, with the optimal growth range being between 7°C and 12°C (Lei 1995).

#### 4.1.3 Coverage of Herbaceous Vegetation

Moreover, herbaceous vegetation coverage exceeding 30% promotes the growth of *O. sinensis* . *O. sinensis* primarily inhabits alpine and subalpine meadows. In these habitats, ghost moth larvae feed on the tender roots of herbaceous plants, directly influencing the distribution of *O. sinensis* (Wu et al. 2009). As altitude increases, the abundance of meadow vegetation also increases, which leads to a higher occurrence of *O. sinensis* . Elevations between 4000 and 4700 m, characterized by typical alpine meadow vegetation, host approximately 90% of China's *O. sinensis* production along with numerous species of ghost moths (Yang et al. 2010).

### 4.2 Trends in the Distribution of *O. sinensis* Under Future Climate Change

The research findings indicate that under the non‐dispersal scenario, the suitable habitat for *O. sinensis* is severely compromised in both extreme climate scenarios, posing a critical threat to its survival. This suggests that warming climates may have adverse effects on their habitats, particularly in India, Pakistan, and parts of China. In Pakistan, the suitable habitat for this fungus will be entirely lost, and in India, the area of lost habitat will be approximately seven times greater than the area of gained habitat. Nepal, Bhutan, and Myanmar are expected to experience a net increase in suitable habitats, with gains outweighing losses. Conversely, suitable habitats at higher elevations (above 5000 m) are expected to increase, potentially because these areas will become more hospitable as temperatures rise (Wei et al. 2021).

The shift in the centroid of the suitable habitats for *O. sinensis* , as shown in Figure 10, demonstrates how geometric concepts can effectively track changes in suitable habitats under different climate change scenarios. Since changes in soil and terrain attributes can be ruled out, the shift in the centroid and the shrinkage of the hot spot area for *O. sinensis* suggest that precipitation and temperature are the main factors contributing to the altered dynamics of suitable habitats (Choden et al. 2021; Guo, Zhao, et al. 2021; Wei et al. 2021). In the 2050s, changes in the suitable habitat showed a similar trend in both hot spot areas and centroid shift, with centroids shifting to warmer but drier locations compared to the current climate. In contrast, the centroid shifts showed opposite directions in the 2100s: centroids shifted to drier and colder conditions under SSP1‐2.6 but to relatively warmer and wetter conditions under SSP5‐8.5. According to the response curve, *O. sinensis* is most frequently found when Bio18 ranges from 100 to 300 mm and Bio11 ranges from −10°C to 4°C (Figure 4). With the rise in summer precipitation, some previously unsuitable habitats reach or exceed the maximum precipitation threshold, becoming suitable habitats. By the 2100s, the centroids reach the minimum threshold of winter temperature under SSP5‐8.5, suggesting that *O. sinensis* will expand into areas with lower temperatures.

### 4.3 Implications for Management and Trading

Projected results indicate notable alterations in the suitable habitat areas and economic value of *O. sinensis* in China. These changes could have substantial economic implications, underscoring the necessity for proactive management strategies. In the 1980s, a kilogram of *O. sinensis* cost 20 Chinese yuan (Liu et al. 2010). By the early 1990s, the price had increased to less than 2000 Chinese yuan. Currently, a kilogram of premium *O. sinensis* can sell for more than 50,000 Chinese yuan (Hopping et al. 2018; Wei et al. 2021). The escalating prices of *O. sinensis* have significantly propelled local economic and social development, transforming some impoverished areas into prosperous ones (Li et al. 2013). In the main producing areas, over 80% of farming and herding families rely on *O. sinensis* to increase their income, with revenue from its sale accounting for 50% to 80% of their total income (Dong et al. 2016; Shrestha et al. 2019; Winkler 2008). Additionally, the influx of a large number of purchasing agents into the production areas has stimulated the development of local industries, including catering, retail, accommodation, and transportation.

China is the world's largest producer of *O. sinensis* , with the majority of its production concentrated in the provinces of Sichuan, Tibet, and Qinghai Province. According to Figure 11, the habitat area, along with the corresponding production and output value in these provinces, is projected to increase under SSP1‐2.6 but decrease under SSP5‐8.5. This reveals that if the world shifts gradually toward a more sustainable path (SSP1‐2.6), the suitable habitat for *O. sinensis* will expand, leading to a production increase of 0.2%–5.2%. Conversely, if resource‐ and energy‐intensive lifestyles are adopted globally (SSP5‐8.5), the main areas of suitable habitat will shrink compared to the baseline condition, resulting in a production decrease of 0.5%–7.2% (Guo, Zhao, et al. 2021).

In addition to climate change, anthropogenic overharvesting significantly accelerates the loss of suitable habitat and disrupts trading practices (Hopping et al. 2018). Due to the challenges in quantifying anthropogenic influences, our composite modeling approach did not account for the impact of human activities on the distribution of *O. sinensis* . As a result, the predicted distribution range of *O. sinensis* may be overestimated. Governmental authorities must formulate and implement scientifically grounded conservation measures promptly. In 2021, *O. sinensis* was designated as a second‐level nationally protected key plant in China. Recently, both national and local governments have implemented a series of protective regulatory measures, including the requirement for a collection permit to harvest nationally protected plants, the delineation of prohibited, controlled, and harvesting zones, and the enforcement of accountability systems (Hu et al. 2005; Tsering and Li 2012; Zhang 2020).

## 5 Conclusions

Our results indicate that altitude is the primary factor influencing the distribution of *O. sinensis* , with a trend toward expansion into higher‐altitude areas in the future. Under a low‐emission scenario (SSP1‐2.6), suitable habitats are expected to slightly increase, with a projected rise of 0.14% by the 2050s and 0.65% by the 2100s. Corresponding production is estimated to increase by 0.2% to 5.2%. In contrast, under a high‐emission scenario (SSP5‐8.5), suitable habitats are projected to decrease by approximately 4.32% by the 2050s and 5.34% by the 2100s, with production expected to decline by 0.5% to 7.2%.

The response of suitable habitats to future climate change varies. This study is helpful for further exploring the future changes in *O. sinensis* habitats and the economy of its main production areas, providing a reference for future conservation efforts. This will contribute to its sustainable utilization and protection.

## Author Contributions

**Liangliang Chen:** data curation (equal), software (equal), writing – original draft (equal). **Hongfen Teng:** conceptualization (lead), funding acquisition (equal), methodology (equal), writing – review and editing (equal). **Songchao Chen:** formal analysis (equal), writing – review and editing (equal). **Yin Zhou:** writing – review and editing (equal). **Dan Wan:** funding acquisition (equal). **Zhou Shi:** project administration (equal).

## Conflicts of Interest

The authors declare no conflicts of interest.

## Supporting information


Table S1.

## Figures and Tables

**FIGURE A1:**
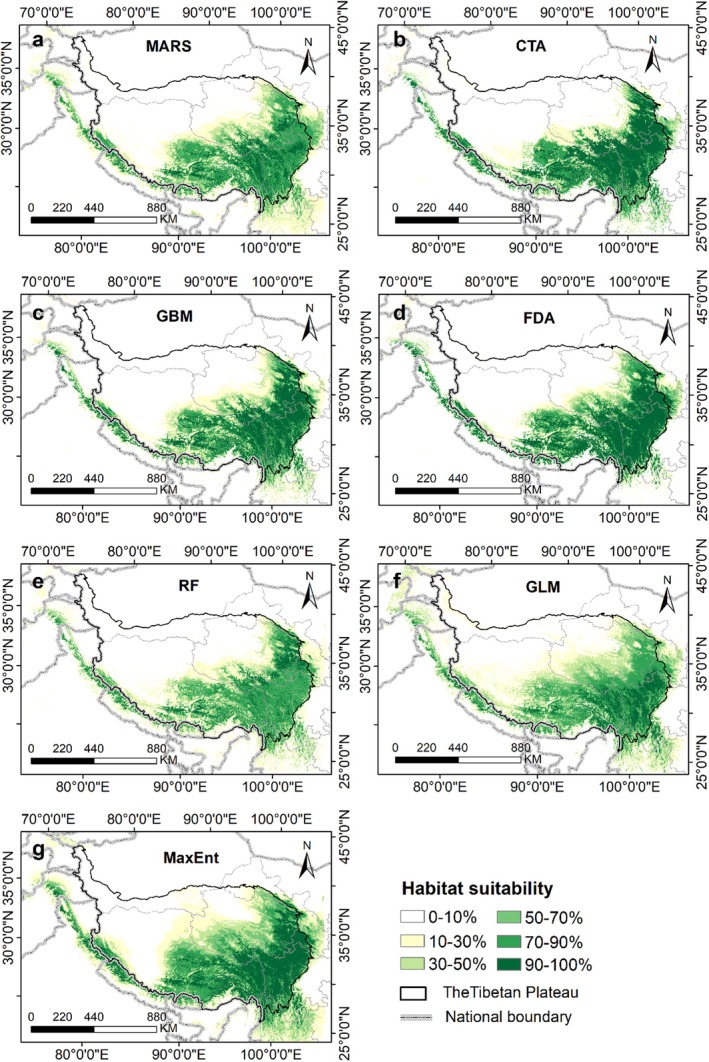
Habitat suitability of O. sinensis based on different models. (a) MARS; (b) CTA; (c) GBM; (d) FDA; (e) RF; (f) GLM; (g) MaxEnt.

**FIGURE A2:**
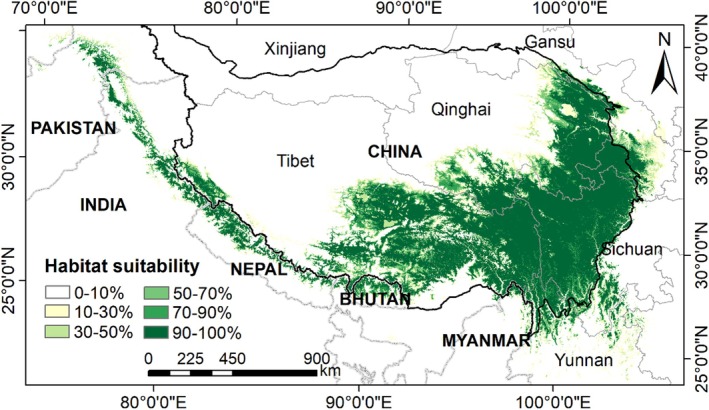
Habitat suitability of *O. sinensis* based on the ensemble model.

**FIGURE B1:**
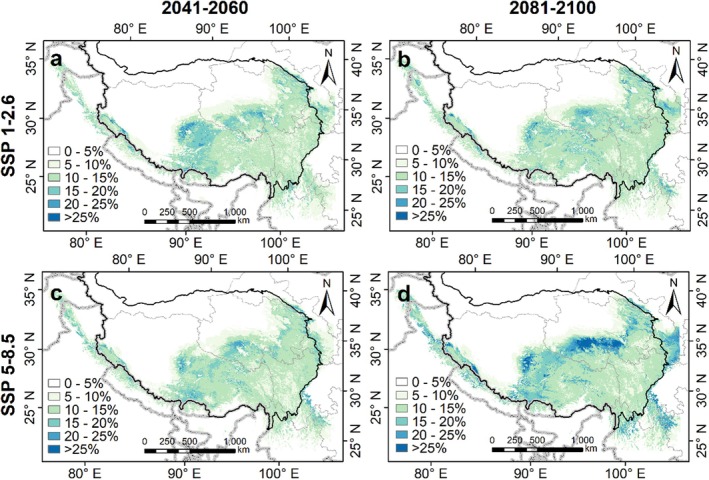
Standard deviation of habitat suitability of *O. sinensis* under different climate scenarios. (a) SSP1‐2.6 in 2041–2060; (b) SSP1‐2.6 in 2081–2100; (c) SSP5‐5.8 in 2041–2060; (d) SSP5‐8.5 in 2081–2100.

## Data Availability

The data that support the findings of this study are available in the Supporting Information and Appendices A and B of this article.

## Appendix A

Habitat suitability of *O. sinensis* based on the selected seven models was shown in Figure A1. According to the simulation results, the predictions from the seven models exhibit high consistency. Regions with high suitability are primarily located in the southeastern part of the Tibet Autonomous Region, the southeastern part of Qinghai Province, the Qilian Mountains region bordering Gansu Province, the western part of Sichuan Province, and adjacent to the southern boundary of the TP in Nepal, Bhutan, and northern parts of India.

Although the consistency among the results of various models was relatively high, there were some differences in detail, particularly in the sizes of high suitability distribution areas. The CTA and MaxEnt models exhibited larger areas of high suitability distribution, primarily concentrated in the border areas of Sichuan Province, Tibet Autonomous Region, and Gansu Province (Figure A1b,g). In comparison, the areas of high suitability distribution predicted by the RF and GLM models were relatively smaller (Figure A1e,f). Regarding the classification of model algorithms, the regression models MARS and GLM showed a more diverse distribution of suitable habitats (Figure A1a,f). Among the classification models of CTA, FDA, RF, and MaxEnt, the MaxEnt model had the largest predicted results. Within the machine learning algorithms of GBM and RF, the spatial simulation results were relatively convergent.

The suitability of *O. sinensis* habitats derived from the ensemble model built with the above seven single models was shown in Figure A2. According to Figure A2, high suitability habitats for *O. sinensis* are mainly concentrated in the southeastern region of the TP and the Himalayan Mountain range. Urban areas with high suitability habitat distribution include Garzê Tibetan Autonomous Prefecture, Qamdo Prefecture, Nagqu Prefecture, Nyingch Prefecture, Aba Tibetan‐Qiang Autonomous Prefecture, Yushu Tibetan Autonomous Prefecture, and Guoluo Tibetan Autonomous Prefecture, as well as Hainan Tibetan Autonomous Prefecture and Haibei Tibetan Autonomous Prefecture. Additionally, some high‐suitability habitats are distributed in Nepal and Bhutan. Low‐suitability habitats are mainly distributed on the outer edges of high‐suitability habitats.

## Appendix B

Figure B1 shows the standard deviation of habitat suitability for *O. sinensis* under different climate change scenarios for various future periods, exhibiting relatively high consistency. The standard deviations are relatively small in the eastern Tibet Autonomous Region, parts of Sichuan within the TP, and areas along the border of Qinghai Province and Sichuan Province, indicating a more stable and accurate prediction of suitable habitats for *O. sinensis* in these regions in the future. These regions show even smaller standard deviations by 2100s under the SSP5‐8.5 scenario. Regions with larger standard deviations are mainly distributed in the central and peripheral areas of the TP, including the central Tibet Autonomous Region and southern Qinghai Province.
